# Understanding the Mechanism of Electrochemical CO_2_ Capture by Supercapacitive Swing Adsorption

**DOI:** 10.1021/acsnano.4c10931

**Published:** 2025-01-17

**Authors:** Grace Mapstone, Tim M. Kamsma, Zhen Xu, Penelope K. Jones, Alpha A. Lee, Israel Temprano, James Lee, Michael F. L. De Volder, Alexander C. Forse

**Affiliations:** † Yusuf Hamied Department of Chemistry, University of Cambridge, Lensfield Road, Cambridge CB2 1EW, U.K.; ‡ Institute for Theoretical Physics, Department of Physics, Utrecht University, Utrecht, CC 3584, the Netherlands; § Mathematical Institute, Department of Mathematics, Utrecht University, Budapestlaan 6, Utrecht, CD 3584, the Netherlands; ∥ Department of Physics, Cavendish Laboratory, University of Cambridge, JJ Thomson Avenue, Cambridge CB3 0HE, U.K.; ⊥ CICA - Interdisciplinary Center for Chemistry and Biology, University of A Coruña, A Coruña 15071, Spain; # Cambridge Display Technology Ltd, Unit 12 Cardinal Park, Cardinal Way, Godmanchester PE29 2XG, U.K.; ∇ Institute for Manufacturing, Department of Engineering, University of Cambridge, 17 Charles Babbage Road, Cambridge CB3 0FS, U.K.

**Keywords:** CO_2_ adsorption, supercapacitive swing adsorption, finite-element modeling, porous carbon, mechanism

## Abstract

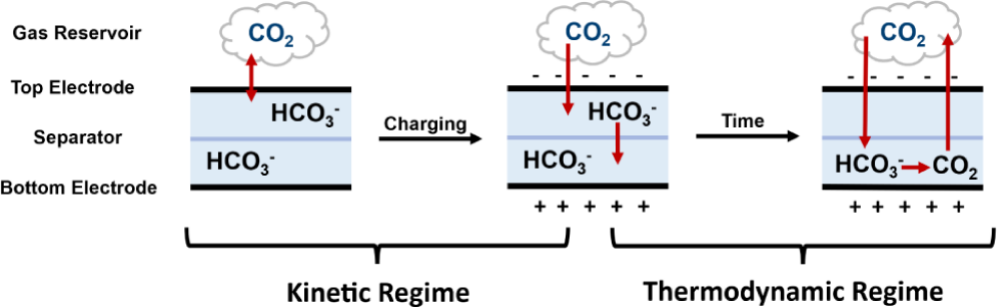

Carbon dioxide capture underpins an important range of technologies that can help to mitigate climate change. Improved carbon capture technologies that are driven by electrochemistry are under active development, and it was recently found that supercapacitor energy storage devices can reversibly capture and release carbon dioxide. So-called supercapacitive swing adsorption (SSA) has several advantages over traditional carbon dioxide capture technologies such as lower energy consumption and the use of nontoxic materials. However, the mechanism for the capture of CO_2_ in these devices is poorly understood, making it challenging to design improved systems. Here, the mechanism of SSA is investigated via finite-element modeling with COMSOL of aqueous continuum transport equations, coupled to the CO_2_ to bicarbonate reaction. This simple computational model reproduces the key experimental observations and shows that charging leads to bicarbonate depletion (or accumulation) in the electrodes, driving CO_2_ capture (or release) at the gas-exposed electrode. This suggests that relevant aspects of the mechanism are captured without excluding other mechanisms that might be at play in parallel as well. At very low charging currents, both experiments and modeling reveal a decrease in the amount of carbon dioxide captured, suggesting the presence of competing processes at the two electrodes, and that SSA is an inherently kinetic phenomenon. This study highlights the importance of the operating conditions of these devices and may aid their development in the future.

The Intergovernmental Panel for Climate Change (IPCC) showed the importance of limiting the increase of the average global temperature to less than 1.5 °C compared to preindustrial temperatures to prevent climate disasters.^1^ There is a global effort, under the Paris Agreement, to reach net zero emissions by 2050.^2^ Investigation into decarbonizing existing industrial processes^3^ is ongoing but for hard to abate sectors, carbon dioxide capture is essential to meet the net zero targets.

Carbon dioxide capture at its most basic form is the selective removal of CO_2_ from a mixture of gases. Current technology uses amines to react with CO_2_ to form carbamates. CO_2_ is then released by heating the carbamates to break the covalent bond with the nitrogen.^4^ The amine-based technology has high adsorption capacities and good selectivity for CO_2_ but there is a large energy demand to reverse the process meaning new technologies are needed.^5^ This led to recent interest in electrochemical carbon capture.^6^ Instead of relying on temperature swings to reverse the capture of CO_2_, and regenerate the sorbent, an electrochemical swing is applied by changing the potential difference in an electrochemical cell. This can range from using redox-active molecules such as quinones to reversibly bind to CO_2_^7^ to electrochemically mediated pH swings using molecules such as phenazines^8^ or Neutral Red.^9^ As the process can be driven solely by renewable electricity, this can further decrease the environmental footprint of the operation of electrochemical carbon capture.

Supercapacitive swing adsorption (SSA) is a promising example of electrochemical carbon capture.^10^ Here, a potential difference is applied between two porous carbon electrodes in an aqueous electrolyte. As the charge of the electrodes is changed, the supercapacitor reversibly captures or releases CO_2_. The system is usually made up of two symmetric conductive carbon electrodes, a separator, and an aqueous electrolyte, typically with 1 M NaCl _(aq)_ as the supporting electrolyte.^10^ The main limitation of this approach is the roughly 8-fold decrease in adsorption capacity (∼100 mmol kg^–1^ normalized to the mass of the gas exposed electrode) as compared to traditional amine technology.^11^ To increase the adsorption capacity of the system several parameters have been investigated including electrolyte concentration,^12^ electrolyte composition,^13^ the structure of the carbon electrodes,^14,15^ the voltage window applied,^16^ the charging protocols^17^ and the polarity of the supercapacitor with respect to the gas reservoir.^11^ While larger adsorption capacities have now been reported, these come at the expense of lower Coulombic and energy efficiencies showing further investigation is still required.^18^

One area of SSA that has not been investigated in detail is the mechanism of CO_2_ capture. Landskron *et al.* initially proposed three mechanisms to explain how supercapacitors may capture CO_2_. First, the gas–solid mechanism assumes that CO_2_ is captured in the empty pores of the carbon electrodes upon charging (i.e., pores not filled with electrolyte take up CO_2_). Second, the molecular liquid–solid mechanism proposes that neutral CO_2_ is captured in the electrolyte-filled pores of the carbon electrodes upon charging. Finally, the ionic liquid–solid mechanism suggests that the CO_2_ forms negatively charged bicarbonate ions that are stored in the electric double layer in the electrolyte at the positive electrode upon charging.^12^ This latter process can occur because CO_2_ is in equilibrium with carbonate and bicarbonate in the presence of water (eq 1).
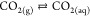
1
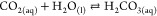
2
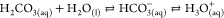
3
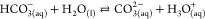
4

These proposed mechanisms can explain some but not all SSA experimental observations. Most importantly, it was shown that the relative charge on the electrode closest to the gas reservoir affects the adsorption behavior of the device.^11,15^ Specifically, it was found that when the supercapacitor was charged in a negative charging mode, i.e., with the gas-exposed electrode charged negatively, carbon dioxide was captured by the cell. In contrast, when the cell was charged in a positive charging mode, i.e., with the gas-exposed electrode charged positively, carbon dioxide release was observed. These findings cannot easily be explained with the three mechanisms described above and highlight a clear need for further investigation into the mechanism of SSA which addresses the CO_2_ speciation and how this changes in different parts of the cell during charging/discharging.

This work therefore investigates the mechanism of carbon capture by SSA through combining results from COMSOL modeling and experimental electrochemical CO_2_ sorption measurements. Our results indicate that the mechanism for SSA is dependent on bicarbonate depletion (or accumulation) in the electrodes to drive the capture (or release) of CO_2_ at the gas-exposed electrode. Upon exploring different charging conditions, we discovered an unexpected decrease in the amount of carbon dioxide captured at lower charging current densities. This suggests competing processes at the two electrodes of the supercapacitor and confirms that SSA is an inherently kinetic phenomenon. A thorough understanding of the mechanism of SSA allows for tailored development of this technology to increase the adsorption capacity and make SSA more competitive with the industry standard amine scrubbing.

## Results and Discussion

### Key Experimental Observations for SSA

**Figure 1:**
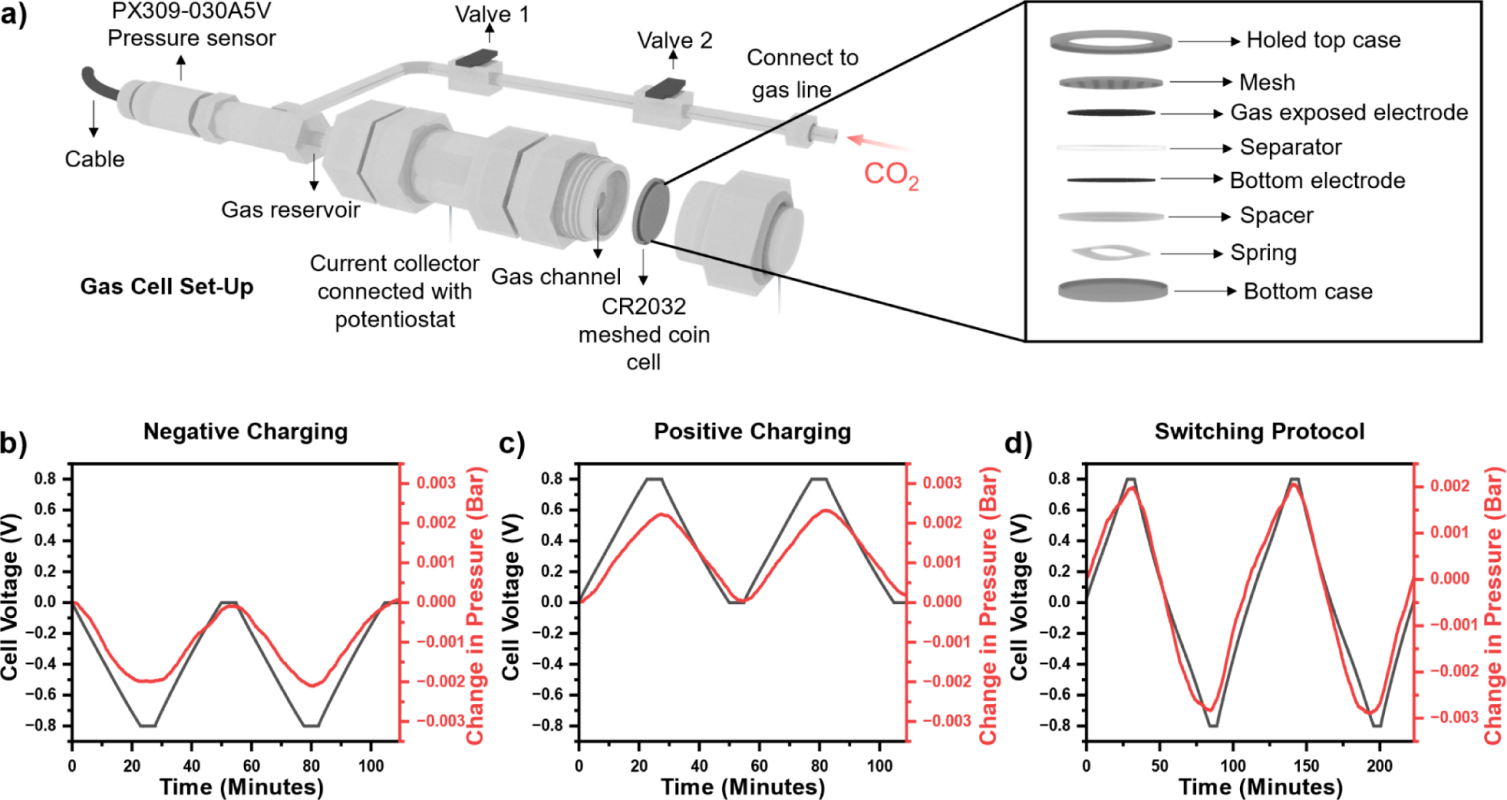
Key experimental observations for SSA. (a) A schematic of the gas cell used and graphs of the effect of changing the cell voltage on the pressure for three different charging protocols: (b) negative charging, (c) positive charging, and (d) switching protocol with a charging rate of 30 mA g^–1^ (in reference to the mass of the gas exposed electrode) and a voltage hold of 5 min. See Figure S2 for the full data set.

**Figure 2:**
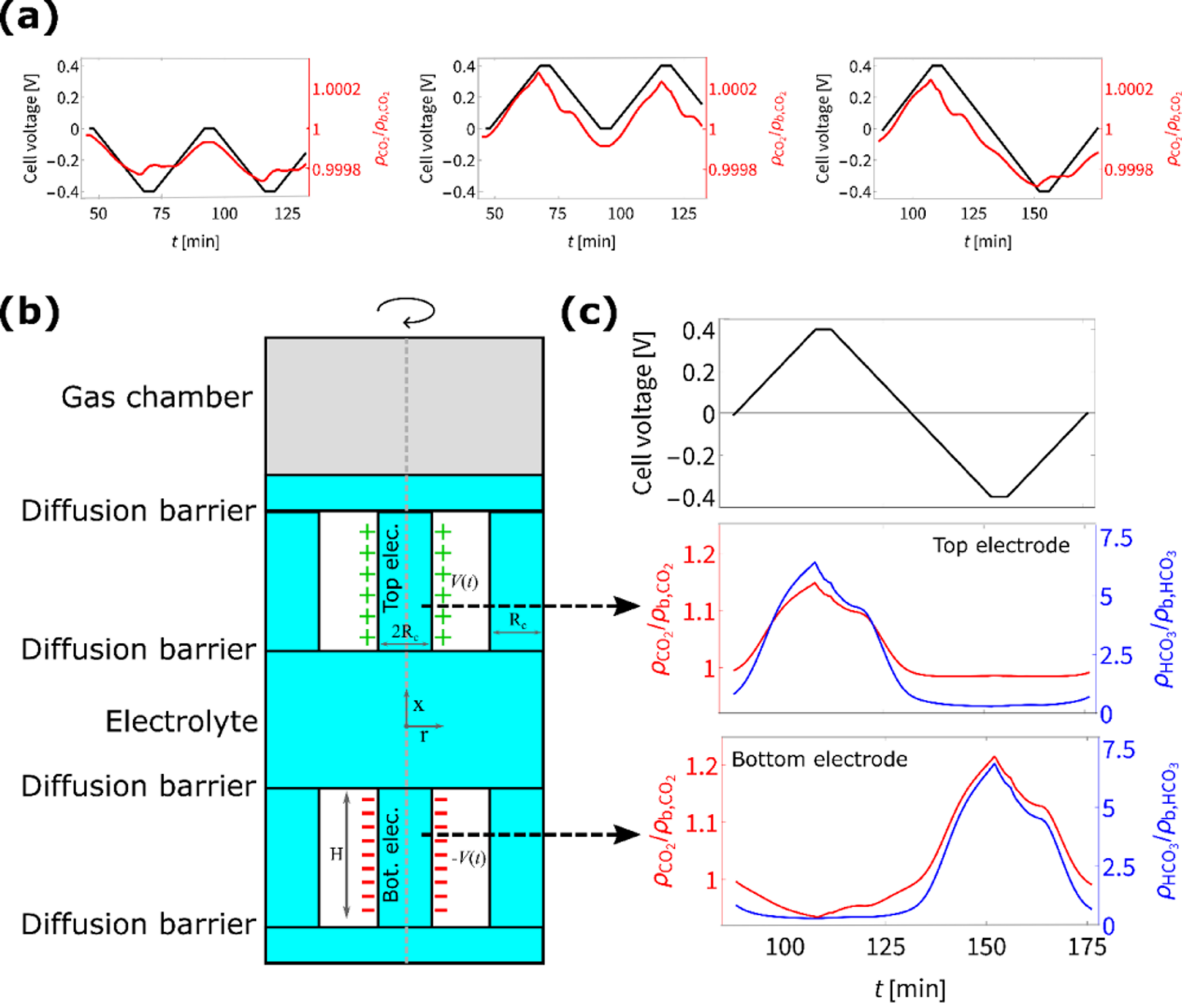
(a) Finite-element calculations using COMSOL for the normalized change in CO_2_ with time for negative, positive, and switching charging regimes moving from left to right, respectively, when charged to ±0.4 V with a voltage hold step of 4 min. (b) A schematic of the model geometry and (c) the change in concentration of CO_2_ (red) and bicarbonate ions (blue) in the top and bottom electrode during one switching protocol cycle.

**Figure 3:**
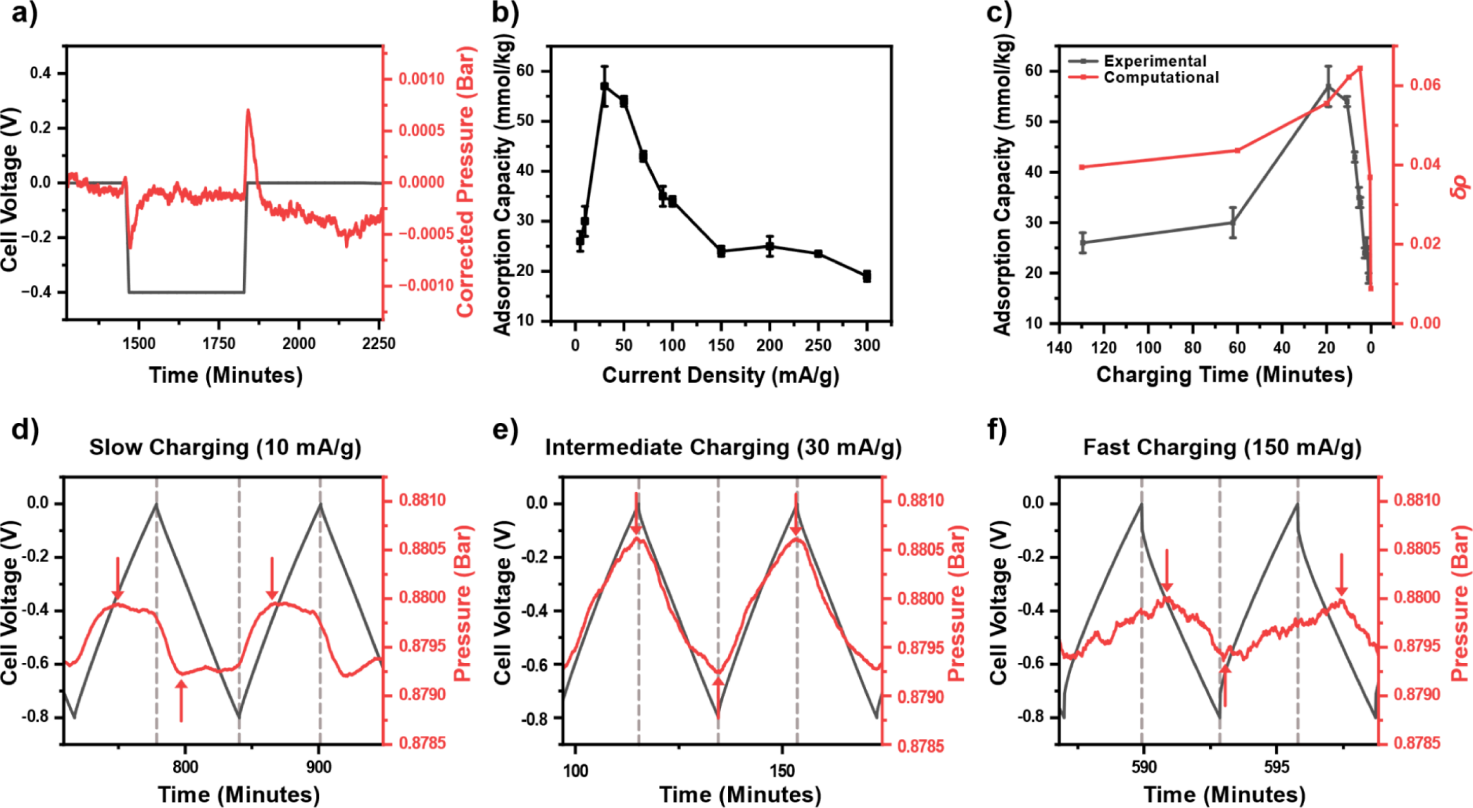
SSA experiments at a wider range of conditions: (a) the effect of a 6-h voltage hold with a current density of 30 mA g^–1^ (in reference to the mass of the gas exposed electrode) on the pressure for the negative charging protocol with the pressure corrected to account for the corrosion and normalized (full data shown in Figure S5), (b) the effect of varying the current density for the negative charging protocol on the adsorption capacity of the system, and (c) a comparison of experimental and computational results for varying the charging time for the negative charging protocol. The pressure and voltage profile for charging at (d) 10 mA g^–1^, (e) 30 mA g^–1^, and (f) 150 mA g^–1^.

Here we explore the impact of three different charging protocols on the adsorption capacity of SSA, similar to previous work.^11^ These are referred to as (1) “negative charging” where the gas-exposed electrode (Figure 1a) was negative for the duration of the cycle (Figure 1b), (2) “positive charging” where the gas-exposed electrode was positive for the duration of the cycle (Figure 1c), and (3) “switching” where the negative and positive charging protocols were combined to take advantage of the full potential window (Figure 1d).^11^ Three-electrode electrochemistry measurements of the supercapacitors under air, nitrogen and carbon dioxide are shown in Figure S1. For the purpose of this investigation, a static gas reservoir of pure CO_2_ was used for our experiments to simplify the system and allow for the elucidation of the mechanism.

The charging mode significantly affects the carbon dioxide capture behavior of the system (Figures 1b–d and S2 for the full data set). In the negative charging mode, carbon dioxide capture takes place when the gas-exposed electrode is subjected to negative potentials (as seen by a decrease in the pressure), while carbon dioxide release occurs during discharge (Figure 1b). In contrast when in the positive charging mode (Figure 1c), carbon dioxide release occurs when the gas-exposed electrode is subjected to positive potentials, and carbon dioxide capture occurs during discharge. These measurements show that the asymmetry in the design of the gas cell, where a single electrode is preferentially exposed to the gas reservoir, manifest in an asymmetry in the behavior of the device. Since the supercapacitor studied here is a symmetric device (with the exception of one electrode being preferentially exposed to gas), the data implies that SSA is a nonequilibrium effect. Indeed, at thermodynamic equilibrium, the same CO_2_ capture (or release) behavior would be expected for both the negative and positive charging modes, which is not what is observed (Figure 1b,c).

An alternative statement of the above results can be made to unify the observations under different charging conditions. Namely when the supercapacitor is charged in a negative direction (i.e., the gas-exposed electrode obtains electrons), the capture of CO_2_ occurs. Conversely, as the supercapacitor is charged in a positive direction, CO_2_ is released. Indeed, the switching protocol (Figure 1d), reverses the supercapacitor polarity and thereby leads to a larger adsorption capacity.^11^ The CO_2_ capture and release is related to the voltage polarity, which indicates the dominant role of the CO_2_-derived bicarbonate species with negative charges in the CO_2_ capture and release processes.

The adsorption capacities for the experiment shown in Figure 1 are 60 ± 2, 68 ± 2, and 150 ± 11 mmol kg^–1^ (mass of the gas exposed electrode) for the negative, positive, and switching charging protocols respectively, similar to previous studies of SSA for this carbon.^11,15^ Additional performance metrics are given in Table S1.

Overall, the findings in Figure 1b–d show the same trends as those reported by Binford *et al.* despite the change in electrolyte from 1 M NaCl_(aq)_ to 1 M Na_2_SO_4 (aq)_.^11^ The change of electrolyte was implemented to reduce the impact of corrosion from chloride ions to the stainless steel in the electrochemical gas cell and meshed coin cell.^19^

### Modeling SSA with Finite-Element Calculations

To help understand part of the mechanism that facilitates the carbon capture features of our device we construct a simple computational model that captures the rough features of the experimental system (see Methods for full details) by calculating the transport of CO_2_ and HCO_3_^–^ in an aqueous electrolyte consisting of nonreactive ions of valency ±1 with equilibrium concentration 100 mM. This computational model incorporates continuum transport equations that include Fickian diffusion, Ohmic conduction, and a source-sink term as a result of the chemical reaction
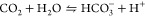
5

Therefore, this model investigates the aforementioned ionic liquid–solid mechanism. We also investigated the effect of volume exclusion through steric effects. Near the electrodes concentrations can get extremely high and thus volume exclusions do locally affect the microscopic double layer structure, but overall similar SSA results are found in both instances so for simplicity we present the results without steric effects here. There are other proposed SSA mechanisms^12^ that would require different physics to be investigated and are not incorporated in our current model. The pH of the system once in contact with CO_2_ cannot be measured experimentally due to the low volume of electrolyte. Additionally the carbon is known to adsorb (or desorb) protons^20^ and therefore the behavior of H^+^ especially near the surface is not entirely known. For this reason, a constant pH of 6 and corresponding H^+^ concentration is chosen within the model, leaving a more detailed approach for future investigations.

In Figure 2 we show the CO_2_ concentration in the modeled gas chamber for the three different charging protocols (Figure 2a), a schematic depiction of the model geometry (Figure 2b), and CO_2_ and HCO_3_^–^ concentrations inside the two modeled electrodes during one full switching protocol cycle (Figure 2c). The concentrations in Figure 2a,c are normalized by their respective equilibrium concentrations ρ_b_.

In Figure 2a we see that the computational model reproduces the characteristic features found in the experiment of CO_2_ adsorption for negative charging, desorption for positive charging and a combination with improved performance for the switching protocol. In order to quantitatively compare the CO_2_ pressure and concentration results of experiments and simulation respectively, we introduce δρ. This is a dimensionless CO_2_ concentration inside the gas chamber that measures the relative change of CO_2_, but also incorporates the volume ratio of the gas chamber compared to the volume of the electrolyte and the voltage ratio of the voltages in the experiments and model (full details in Methods). The differences in applied voltages are due to the model becoming unstable at the higher voltages used in the experiments. We find that the experiments display a total maximal change δρ of 0.25, 0.29, and 0.62 for negative charging, positive charging and switching respectively, whereas the computational model predicts a maximal δρ of 0.065, 0.10, and 0.15 respectively. Precise comparisons are not fully warranted due to the coarse nature of the model, but that the results are well within an order of magnitude does support that the method we propose in this work is at least partially responsible for the observed carbon capture features exhibited by the device.

The above-mentioned results follow from a model that tries to capture the device’s essential features while otherwise remaining as simple as possible, schematically shown in Figure 2b. To bridge the length scales ranging from nm inside the pores to mm for the overall device, we let a single nanochannel (middle channel in Figure 2b) of radius *R*_c_ = 2.5 nm and length *H* = 50 nm represent the complex porous structure of an electrode. Diffusion barriers at either end of the nanochannels approximate the mm device features and slow down diffusion via a simple linearized diffusive flux over a distance of 0.5 mm. The effective thickness of the computational model is thicker than the physical device, but the actual lengths of the pathways can also vary considerably in the porous electrodes. Therefore, we chose the thickness of 0.5 mm for simplicity. Uncharged pathways (left and right channels in Figure 2b) are also present alongside the charged nanochannels to model the larger mesopores in each electrode, whose charge screened surfaces do not affect the majority of the internal volume due to their larger radii, rendering them effectively uncharged. Lastly a gas chamber is in contact with the electrolyte at one side of the system, which can freely exchange CO_2_ with the electrolyte. The entire system is azimuthally symmetric around its central axis.

To try and rationalize the behavior of CO_2_ inside the gas chamber shown in Figure 2a, we show in Figure 2c, for one period of a switching protocol voltage sweep (top), the CO_2_ and bicarbonate concentration inside the top electrode (middle) and the bottom electrode (bottom) normalized by their respective equilibrium concentrations ρ_b_. We see that a positive (negative) cell voltage results in bicarbonate accumulation (depletion) in the electrode. This shifts the equilibrium position (eqs 1 and 2), resulting in an indirect change in the CO_2_ concentration. When the top channel is depleted of bicarbonate (and consequently of CO_2_), then CO_2_ can diffuse into the channel from the gas chamber, effectively adsorbing CO_2_. This also explains why CO_2_ is depleted more strongly in the bottom channel, since here there is no direct influx of CO_2_ from the gas chamber. When bicarbonate accumulates in the top channel, then an excess of CO_2_ is produced, resulting in a CO_2_ flux into the gas chamber, thereby desorbing the CO_2_.

As can be seen in Figure 2a, the CO_2_ starts to revert back while the voltage is still held. Here the charged channel that is not in direct contact with the gas chamber counteracts the channel that is in contact with the gas chamber, be it in a delayed manner due to the larger separation to the gas chamber. The experiments also support this as the CO_2_ concentration reverts back after longer waits as we will show in the next section.

Our model supports that SSA is at least partially driven by the accumulation or depletion of bicarbonate ions in the electrodes upon charging. When the gas-exposed electrode is the positive electrode (positive charging mode), then this leads to an overaccumulation of CO_2_ in that electrode, and CO_2_ release occurs. In contrast, when the gas-exposed electrode is the negative electrode (negative charging mode), depletion of bicarbonate and therefore CO_2_ sets up a driving force for CO_2_ capture. However, the model is not a full simulation of the experimental device, but rather a coarse representation that features considerable simplifications with respect to the actual device’s features, and only incorporates continuum transport equations and the CO_2_-bicarbonate reaction. Therefore, there are possible mechanisms not taken into account in our current approach. Nevertheless, these results do hint toward the ionic liquid–solid mechanism as an important contributor to the observed carbon capture features.

### Testing the Limiting Behavior of the System

As discussed above, at thermodynamic equilibrium we would anticipate no difference in the CO_2_ capture behavior under positive and negative charging modes. Our proposed mechanism above also suggests that at thermodynamic equilibrium, less or potentially even zero CO_2_ may be captured. Indeed, at long time scales, the CO_2_ gas would have enough time to diffuse through the whole supercapacitor system and create a pseudosymmetric system where neither of the electrodes is more gas exposed than the other. This would create the conditions for the capture and release of CO_2_ at the same time. To test this hypothesis, SSA experiments were carried out with much longer voltage holds with a duration of 6 h. Here, a voltage window of −0.4 to 0 V was used to reduce the impact of corrosion to the stainless-steel coin cell components, which is exaggerated when using longer voltage holds (Figure S4).

Figure 3a, shows that holding the cell voltage for longer results in no apparent net capture of CO_2_. Initially, as the supercapacitor is charged in a negative direction, there is the expected pressure drop indicating the capture of CO_2_. Then within an hour, this captured CO_2_ begins to desorb and the pressure returns to an apparent equilibrium position. A similar phenomenon then occurs when charging in a positive direction back to 0 V (Figure 3a). Initially, there is the release of CO_2_ and then at longer times the CO_2_ is captured again with the pressure returning to the same apparent equilibrium position. These findings contrast significantly with the much faster SSA experiments in Figure 1b–d, and show that it is the direction of current flow that determines whether CO_2_ is captured or released. The same trends are seen for both the positive and switching charging protocols (Figure S5) with the experiment repeated a total of three times to ensure a reproducible result (Figures S5–S7).

This reversibility shows that when the system remains at one cell voltage for long enough, the distinction between the top and bottom electrode diminishes creating a pseudosymmetric state as we hypothesized. This creates the conditions for the capture and release of CO_2_ to occur simultaneously at the two different electrodes, resulting in the establishment of thermodynamic equilibrium at longer time scales. This effect happens regardless of which direction the system is charged in. The longer voltage holds show that the capture (or release) of CO_2_ only occurs in a short time frame, confirming that SSA is a kinetic effect driven by the kinetic CO_2_ capture differences between the gas-exposed electrode and the bottom electrode. At extended times, when the system is under thermodynamic control, the net capture of CO_2_ becomes negligible.

To understand the trade-off between thermodynamic and kinetic effects, a series of SSA experiments and simulations were carried out in which the current density was varied in the negative charging mode (Figure 3b,c) and the trends in the pressure curves were examined closely (Figure 3d–f). The experimental measurements revealed a maximum CO_2_ adsorption capacity of 57 ± 4 mmol kg^–1^ at 30 mA g^–1^ (Figure 3b). At this current density, the variation of the CO_2_ pressure is synchronous with the device voltage (Figure 3e). However, if the supercapacitor is charged more slowly, with current densities below 30 mA g^–1^, then a reduction in the adsorption capacity occurs (Figure 3b). The same trends were reported by Xu *et al.* when YP80 activated carbon was used as the electrode material^15^ and by Zhu *et al.* when BPL 4 × 6 carbon was used.^17^ This indicates that this behavior is consistent across different activated carbon electrodes.

When the supercapacitor is charged slowly, the peak in the adsorption and desorption shifts to the left-hand side, meaning that the peak is reached during the charging step itself (as indicated by the red arrows in Figure 3d). As this peak is reached sooner, there is enough time for some reversibility to occur, and the captured CO_2_ begins to release again or vice versa when discharging the supercapacitor. This matches the behavior seen under long voltage holds (Figure 3a) and causes the decrease in adsorption capacity at slow charging conditions (Figure 3b). Indeed, as the system is charged more slowly, there is sufficient time for the system to shift into the thermodynamic regime. Thus, even as the system is perturbed through charging (or discharging), there is time to re-equilibrate toward the thermodynamic state. At the extreme, if the system was charged slowly enough, we anticipate there would be zero (or very little) net capture of CO_2_ as any change to the equilibrium position (eq 1) would have enough time to re-equilibrate before any capture of CO_2_ was measured.

If the supercapacitor is charged faster with current densities greater than 30 mA g^–1^ then there is a decrease in the adsorption capacity (Figure 3b). For this faster charging regime, the peak or trough of the pressure curves shift toward the right-hand side showing a delayed response of the pressure curve compared to the change in the cell voltage (as indicated by the red arrows in Figure 3f). This indicates that the whole system, including both electrodes, is becoming mass transport limited where the change in the voltage is too fast for the slow-moving diffusion of CO_2_ and bicarbonate ions. This limits the adsorption capacity causing its decrease at faster current densities. The system reaches a plateau at around 150 mA g^–1^ which is consistent with reaching a mass transport limited system.

This behavior was modeled using the same computational model as in Figure 2b, the results of which are shown in Figure 3c. A reduction in adsorption capacity emerges for both short and long charging times in the mass transport limited and thermodynamic regimes respectively, similar to the experiments. For short charging times CO_2_ is still flowing into the gas chamber from the gas-exposed electrode while the cell voltage is already at −0.4 V (the minimal voltage in the model), i.e., the system is mass transport limited (Figure S8a). With a charging time of ∼5–20 min there is little CO_2_ flux going from the gas chamber into the gas-exposed electrode once the cell voltage is at its minimal value of −0.4 V (Figure S8b), showing that the adsorption is near its maximum as we indeed see in Figure 3c. For the long charging time of 130 min there is now instead CO_2_ flux going into the gas chamber from the gas-exposed electrode, originating from the electrode that is not exposed to the gas (Figure S8c). This reversibility is a delayed response to the increase in CO_2_ concentrations in the bottom electrode, thereby counteracting the initial adsorption, as we indeed hypothesized for the thermodynamic regime. However, quantitatively there are some differences between the model and experiments (Figure 3c). This can possibly be explained by the coarse minimalistic features of the model, which do not emulate the full detail of the device and only incorporates continuum transport of ionic and chemical species with one possible chemical reaction, thereby foregoing any other possible physical mechanism that might also be at play here. Therefore, the further investigation of kinetic and thermodynamic effects in SSA are important direction for future investigations, both computationally and experimentally.

Summarizing, the relationship between the current density and the CO_2_ adsorption capacity is ruled by the trade-off between two competing effects. One is the thermodynamic control when charging slowly and the second is mass transport which limits adsorption capacity when charging fast. This suggests that any SSA set up would have a peak in the adsorption capacity, though the current density that this occurs at would vary between systems. For example, using a flow setup and mixed gases of 15% CO_2_ and 85% N_2_, Landskron *et al.* showed that under the same charging conditions, the current density of 20 mA g^–1^ gave the best performance.^17^ Understanding and exploiting kinetic and thermodynamic effects in SSA is an important direction of future research.

## Conclusion

In this work we have found new insights into the mechanism of SSA with both experiments and computational modeling. The COMSOL model showed that when the supercapacitor is charged in a negative direction, the concentration of bicarbonate ions decreases at the top electrode allowing for more CO_2_ to diffuse in leading to the capture of CO_2_. The converse is true when charging in a positive direction where an increase in the bicarbonate concentration at the gas-exposed electrode leads to the desorption of CO_2_. The limiting behaviors of the system were probed to understand the difference in the kinetic and thermodynamic regimes. When the supercapacitor was charged slowly or had a long voltage hold, the capture of CO_2_ was reversible showing that SSA is inherently a kinetic effect and only occurs on shorter time scales. SSA is also limited by the current density where, when charging fast enough, the system becomes mass transport limited. By rational design of the device and materials to allow for faster mass transport of CO_2_ and HCO_3_^–^ ions, faster current densities would be favored thus improving the productivity of the CO_2_ capture. This information can be used to inform future device and material design to increase the performance of SSA.

## Materials and Methods

All materials were used without further purification from suppliers specified below.

### Electrode Fabrication

The electrode film preparation followed the traditional method used for activated carbons.^21^ The electrode films were made with a ratio of YP50F:PTFE of 95:5 wt %. An activated carbon YP50F (200 mg, Kuraray) and ethanol (ca. 2 mL, VWR Chemicals) mixture was sonicated for 10 min. PTFE (60 wt % in water) (17.5 mg, Sigma-Aldrich) was added to a watch glass with a few drops of ethanol and the sonicated solution was added to this. This was stirred by hand for 15 min and then the dry parts were slowly incorporated to form a black material with chewing gum-like consistency. Once all the ethanol had evaporated, this was then kneaded with a spatula for 5 min with drops of ethanol added periodically to prevent the material from drying out. This was rolled out to a thin film and then dried at 100 °C under dynamic vacuum for at least 24 h.

### Three-Electrode Measurements

The three-electrode measurements were conducted in a Swagelok cell as shown in Figure S1b. Two identical carbon electrodes (YP50F, 1/4-in. diameter) were used as the working and counter electrodes, with two separator disks (GF/A Whatman, 5/16-in. diameter) placed in between the electrodes. The cell was assembled horizontally in the Swagelok cell (Figure S1b). For the electrochemistry measurements in air, 1 M Na_2_SO_4 (aq)_ (750 μL) was added and then the Hg/HgO reference electrode was submerged in the electrolyte from the top input (Figure S1b). The supercapacitor was precycled by conducting 20 cyclic voltammetry cycles at a scan speed of 2 mV s^–1^ from −0.8 to +0.8 V. Cyclic voltammetry tests were then conducted on the cell at a scan rate of 1 mV s^–1^. When measured under N_2_ or CO_2_, the same procedure was followed up to precycling. After precycling, more 1 M Na_2_SO_4 (aq)_ (1250 μL) was added. The electrolyte was purged with N_2_ at a flow rate of 10 mL min^–1^ for 20 min. Five cyclic voltammetry cycles were then conducted at a scan rate of 2 mV s^–1^ under continued gas flow. The electrolyte was then purged with CO_2_ at a flow rate of 10 mL min^–1^ for 30 min and a further 5 cyclic voltammetry cycles were performed at a scan rate of 2 mV s^–1^.

### Gas Cell Assembly

Two carbon electrodes were cut with a steel punch (0.5-in. diameter) with a mass ranging between 13.2 to 17.9 mg. The separator (GF/A Whatman) was then cut with a steel punch (5/8-in. diameter). This was then added to the coin cell (CR2032, Cambridge Energy Solutions) from the bottom up as bottom case, conical spring, spacer (0.5 mm), bottom electrode, separator, gas-exposed electrode and then 1 M Na_2_SO_4 (aq)_ (100 μL) was pipetted onto the separator. A stainless-steel mesh (16 mm diameter) and machined holed (12 mm diameter) top case were added before sealing in a Compact Hydraulic Coin Cell Crimper (Cambridge Energy Solutions) at 80 kg cm^–2^. The assembled coin cell was then added to the gas cell with the meshed side facing the gas reservoir (as shown in Figure 1).

### Carbon Dioxide Dosing

To exchange the air in the gas reservoir with CO_2_ (99.80% purity, BOC), a custom gas manifold was used. To prevent evaporation of the electrolyte the cell was exposed to a static vacuum by opening the two valves (Figure 1a) dropping the gas cell pressure to ∼0.45 bar. The valve closest to the cell was then closed and the gas manifold was dosed with CO_2_ at ∼1.4 bar. The reduced pressure in the gas cell ensures that when the valve to the cell is opened, there is a mixing of the gas reservoir with the CO_2_ in the gas manifold. The valve is closed, and the manifold then put back under dynamic vacuum and the process repeated 4 more times to give a 98.7% by volume CO_2_ headspace assuming ideal gas mixing.

### Pressure Measurement

The pressure data was recorded using an Omega PX309-030A5V pressure transducer. All the pressure data present in this paper was smoothed over 10% of the cycle time for a negative charging protocol. This is demonstrated further in Figure S9.

### Electrochemical Measurement

All experiments were carried out on the VSP-3e potentiostat which measured the current, voltage and pressure and recorded values using Biologic software. Before any experiment the gas cell was allowed to reach 30 °C in an incubator oven (SciQuip Incu-80S) for 12 h. Then the supercapacitor was precycled by conducting 20 cyclic voltammetry cycles at a scan speed of 1 mV s^–1^ from −0.8 to +0.8 V. The charging protocols used in this work for the SSA experiments are split into four main sections here. For negative charging: charge the supercapacitor to −0.8 V at a constant current, hold the cell voltage at −0.8 V for 5 min, discharge the supercapacitor back to 0 V at a constant current of the same magnitude as the charge step, and finally hold the cell voltage at 0 V for 5 min. This protocol was also applied for the positive and switching experiments but with different voltage windows. This protocol was also used for the long voltage hold experiments but here with a limited voltage window of ±0.4 V and cell voltage holds of 6 h instead of 5 min. A modified protocol was used for varying the current density where a voltage window of ±0.8 V was used but there was no cell voltage hold applied in between charging/discharging steps to exaggerate the effect of the current density on the results.

### Computational Model

To investigate the mechanism for the capture of CO_2_ in this system, finite-element (FE) calculations using the software package COMSOL were employed (Figure 2). The proposed mechanism involves the transport of molecular and ionic species in an aqueous environment, which we investigate using traditional diffusion-conduction equations in the form of the Poisson–Nernst–Planck equations. In this system of equations the flux ***j***_*i*_ of any of the *n* species *i* is assumed to satisfy:
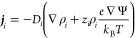
6which includes Fickian diffusion, Ohmic conduction and steric effects due to volume exclusion. Here ψ is the electric potential, *e* the elementary charge, *k*_B_ the Boltzmann constant, *D*_*i*_ is the diffusion coefficient of species *i*, ρ_*i*_ is the concentration, and *z*_*i*_ the valency. In the Supporting Information, we also present results for the same computational model, but where the flux is assumed to satisfy
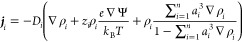
7with *a*_*i*_ the hydration radius. This extra term accounts for volume exclusion through steric effects,^17^ the system of equations using eq 7 is known as the Generalized Modified Poisson-Nernst–Planck (GMPNP) equations. The results in Figure 2 are based on eq 6, while the results in Figure S3 are based on eq 7.

The electric potential is influenced by the space charge density  according to the Poisson equation:
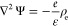
8with ε = 80.23ε_0_ the dielectric constant of water. The creation of depletion of particles due to chemical reactions is of importance here, so particle conservation is balanced with a source or sink term *R*_*i*_ in the continuity equation:
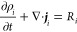
9where the chemical reaction that we take into account is
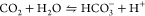
10

In this reaction the conversion of H_2_CO_3_ to HCO_3_^–^ is skipped since this reaction occurs quasi-instantaneous such that the HCO_3_^–^ concentration is divided by the equilibrium constant of this conversion *K*_2_ = 2 × 10^–4^ M to account for this.^22^ The forward reaction rate is 6.6 M^–1^ s^–1^^21^ where the concentration of water is assumed to be 55.6 M and the backward rate is 24.8 s^–1^ where [H^+^]^21^ is assumed to correspond to a pH of 6 to match the experiment. The system of equations is closed upon imposing blocking boundary conditions on all solid walls, ***n***·***j***_*i*_ = 0 with ***n*** the (inward) normal on the walls of the device, ψ = ±*V* at the positive and negative electrode, respectively. We numerically solve the full (GM)PNP equations within the geometry described below using the FE analysis package COMSOL (the mesh used is shown in Figure S10). The initial *V* = 0 at the electrodes and we set ρ_*i*_ to the equilibrium concentrations for all species.

The model features five species of particles, an electrolyte component consisting of nonreactive ions of valency ±1 with equilibrium concentration 100 mM, solvation size 0.8 nm, and diffusion coefficient 1 μm^2^ ms^–1^; CO_2_ with equilibrium concentration 25.8 mM, solvation size 0.23 nm, and diffusion coefficient 1.91 μm^2^ ms^–1^; HCO_3_^–^ with equilibrium concentration 7.65 mM, solvation size 0.8 nm, and diffusion coefficient 1.185 μm^2^ ms^–1^; and H^+^ with equilibrium concentration 10^–7^ M, solvation size 0.56 nm, and diffusion coefficient 9.311 μm^2^ ms^–1^.^23−25^

As we chose to take the simplest possible approach in this initial computational model, we set the concentration of H^+^ to 10^–6^ M to match the pH of 6 in the reaction of eq 9. However, H^+^ is also charged and therefore interacts with the charged electrodes. Within Poisson–Boltzmann theory, in a dilute regime with fully developed double layers, this would actually mean  is a constant within the electric double layers since both ions ∝Exp(±eV/*k*_B_*T*). However, the electrodes are also known to adsorb or desorb H^+^,^20^ additionally we are in a high concentration so locally near the electrodes Poisson–Boltzmann theory is not accurate. Additionally, other reaction pathways including e.g., reactions involving H^+^ could also play a role.^23,26^ Therefore, H^+^ concentration is an interesting next step to more accurately investigate the fascinating device dynamics.

The overall experimental device length scale is of order mm, but it incorporates features of the nm length scale, presenting us with the computational challenge of bridging this disparity in one computational model. Therefore, we let a single nanochannel represent the complex porous structure of an electrode. The mm scale of the device is then approximated by placing diffusion barriers at either end of the nanochannel which slows down diffusion via a simple linearized diffusive flux *j*_*i*_ = −*D*_*i*_,_b_(ρ_*i*_(*x*_*+*_) – ρ_*i*_(*x*_*–*_))/*d*_b_. Here *D*_*i*_,_b_ = 10^–2^*D*_*i*_ to account for the phenomenon that effective self-diffusion coefficients of ions, when situated in a nanoporous material, are decreased by over 2 orders of magnitude compared with bulk electrolyte,^27^ ρ_*i*_(*x*_+_) and ρ_*i*_(*x*_*–*_) are the concentrations of species *i* just above and just below the barrier, respectively, and *d*_b_ = 0.5 mm is the thickness of the barrier. The interface between the electrolyte is also approximated by a diffusion barrier which is only permeable to CO_2_ (i.e., *D_i_*,_b_ = 0 for all other species) and the thickness is set to the small 0.1 nm to ensure that gas and electrolyte concentrations are always in equilibrium at the interface, since this conversion is quasi-instanteneous.^22^ The overall resulting geometry of the computational model is schematically depicted in Figure 2b. Here we see an axisymmetric model with charged nanochannels of radius *R*_c_ = 2.5 nm and length *H =* 50 nm at either side of an electrolyte separator with the top channel in direct contact with a gas chamber. Additional uncharged channels of height *R*_c_ = 2.5 nm are placed to take the effectively uncharged pathways through the macropores in the electrode into account.

To indicate the change of CO_2_ concentration in the gas chamber we introduce the dimensionless parameter
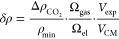
11

Here  is the change in CO_2_ concentration divided by the minimum CO_2_ concentration ρ_min_ during the respective cycle to obtain the relative change in CO_2_, Ω_gas_ is the volume of the gas chamber, Ω_el_ is the volume of the electrolyte, *V*_exp_ and *V*_CM_ are the maximum voltages in the experiment and computational model, respectively. We do remark that the volume ratios are the same for both the experiment and the computational model.

## Data Availability

All raw data files are available in the Cambridge Research Repository, Apollo, at 10.17863/CAM.109172. A version of this manuscript before peer review was added to a preprint server: Grace Mapstone; Tim M. Kamsma; Zhen Xu; Penelope K. Jones; Alpha A. Lee; Israel Temprano; James Lee; Michael De Volder; Alexander C. Forse; Understanding the Mechanism of Electrochemical CO_2_ Capture by Supercapacitive Swing Adsorption. 2024, ChemRxiv, https://chemrxiv.org/engage/chemrxiv/article-details/6777c942fa469535b9a14468 (accessed 01/13/2025).

## Supporting Information Available

Further calculations of key parameters, full experimental data sets including the voltage and pressure profiles and key repeat experiments and mesh used in COMSOL model (PDF)
