# Supplementary material for: Understanding the Mechanism of Electrochemical CO2 Capture by Supercapacitive Swing Adsorption
Source: ACS Nano. 2025 Jan 17;19(4):4242–50. doi: 10.1021/acsnano.4c10931 (PMC11803910; doi:10.1021/acsnano.4c10931)
Supplement: Supplementary file 1 — nn4c10931_si_001.pdf [file nn4c10931_si_001.pdf]

Supplementary Information for:

Understanding the Mechanism of Electrochemical CO_2 Capture by Supercapacitive Swing Adsorption

Grace Mapstone[1], Tim M. Kamsma[2,3,4], Zhen Xu[1], Penelope K. Jones[4], Alpha A. Lee[4], Israel Temprano[1,5], James Lee,[6] Michael F. L. De Volder[7], Alexander C. Forse[1*]

[1]Yusuf Hamied Department of Chemistry, University of Cambridge, Lensfield Road, Cambridge, CB2 1EW, UK
[2]Institute for Theoretical Physics, Department of Physics, Utrecht University, Utrecht 3584 CC, The Netherlands
[3]Mathematical Institute, Department of Mathematics, Utrecht University, Budapestlaan 6, 3584 CD Utrecht, The Netherlands
[4]Department of Physics, University of Cambridge, Cavendish Laboratory, JJ Thomson Avenue, Cambridge, CB3 0HE, UK
[5]CICA - Interdisciplinary Center for Chemistry and Biology, University of A Coruña, 15071, A Coruña, Spain
[6]Cambridge Display Technology Ltd., Unit 12 Cardinal Park, Cardinal Way, Godmanchester, PE29 2XG, UK
[7]Institute for Manufacturing, Department of Engineering, University of Cambridge, 17 Charles Babbage Road, Cambridge, CB3 0FE, UK

* Corresponding author's email: acf50@cam.ac.uk

Table of Contents

Table of Contents 2
Calculations: 3
SI Figures 5
SI Figure S1 5
SI Figure S2 6
SI Figure S3 7
SI Figure S4 80
SI Figure S5 9
SI Figure S6 10
SI Figure S7 11
SI Figure S8 12
SI Figure S9 13
SI Figure S10 14
SI Table 15
SI Table S1 15

Calculations:

The volume of the gas cell varies from cell to cell and so is calculated each time a gas cell is assembled. A pressure change is measured by from evacuation of the space between the two valves which is a known, previously measured volume (Figure 1). Therefore, the following equation can be applied:

$$P_1V_1 = P_2V_2 \qquad 1$$

Where P_1 is the initial pressure recorded, V_1 is the volume of the gas reservoir, P_2 is the pressure recorded after the valve is opened and V_2 is the volume of the gas reservoir plus the volume between the two valves. This can therefore be arranged to give:

$$V_1 = \frac{P_2x}{P_1 - P_2} \qquad 2$$

Where x is the volume between the two valves.

The adsorption capacity (AC) was calculated using:

$$AC = \left(n_{max} - \left(\frac{n_{min-1} + n_{min+1}}{2}\right)\right) \div m \qquad 3$$

Where n_{max} is the maximum value in moles in a given cycle, n_{min-1} is the minimum value in moles to the left of n_{max}, n_{min+1} is the minimum value in moles to the right of n_{max} and m is the mass of the carbon in the gas exposed electrode. The average of the two adjacent minima is used to reduce the error in slight changes to the overall pressure with time most likely due to corrosion.

The coulombic efficiency (CE) was calculated using:

$$CE = \frac{Q_d}{Q_c} \times 100 \qquad 4$$

Where Q_d is the total charge during discharge of the supercapacitor and Q_c is the total charge during the charging of the supercapacitor. For this reason, the coulombic efficiency is not reported for the switching protocol as the supercapacitor is both charged and discharged during the “charging” (and “discharging”) part of one cycle under this protocol.

The resistance (R) is calculated using:

$$R = \frac{\Delta V}{\Delta I} \qquad 5$$

Where ΔV is the change in voltage as a current is applied to the supercapacitor and ΔI is the change in current.

The gravimetric capacitance (C_g) is calculated using:

$$C_g = \frac{m_1 + m_2}{m_1 m_2} \times I \times \frac{dt}{dV} \qquad (6)$$

Where m_1 and m_2 are the masses of the active material in the two electrodes, I is the current, dt is the change in time and dV is the change in voltage.

The electrical energy consumption (EC) is calculated using:

$$EC = \frac{E_c - E_d}{n_a} \qquad (7)$$

Where E_c is the electrical energy consumed during charging of the supercapacitor, E_d is the electrical energy consumed during discharge of the supercapacitor and n_a is the number of mols of CO_2 adsorbed in one cycle.

The errors for all of the above parameters are calculated based on 1.95 standard deviations from the average value across all cycles measured.

SI Figures

SI Figure S1

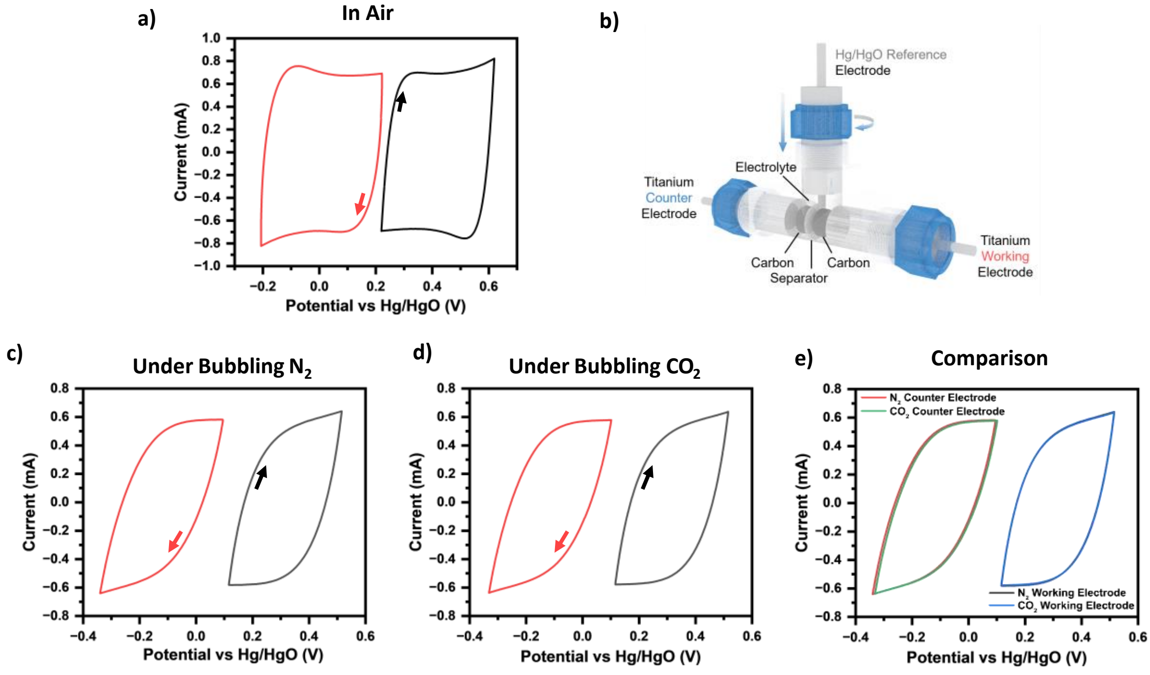

Figure S1: a) A three-electrode measurement with identical YP50F working and counter electrodes with 1 M $Na_2SO_{4\ (aq)}$ electrolyte against a Hg/HgO reference electrode recorded with a scan rate of 2 mV s^{-1}. b) A schematic of the Swagelok cell used to conduct the three-electrode measurements. A three-electrode measurement with identical YP50F working and counter electrodes with 1 M $Na_2SO_{4\ (aq)}$ electrolyte against a Hg/HgO reference electrode were recorded with a scan rate of 2 mV s^{-1} under bubbling the electrolyte with c) nitrogen and d) carbon dioxide. e) A comparison of the three-electrode measurements under bubbling with nitrogen and carbon dioxide. Notes: this three-electrode measurement demonstrates that the measured potential window is slightly larger for counter electrode compared to the working electrode when under air (potential window of 0.400 V and 0.428 V for the working and counter electrode), bubbled nitrogen (potential window of 0.400 V and 0.435 V for the working and counter electrode) and bubbled carbon dioxide (potential window of 0.399 V and 0.433 V for the working and counter electrode).

SI Figure S2

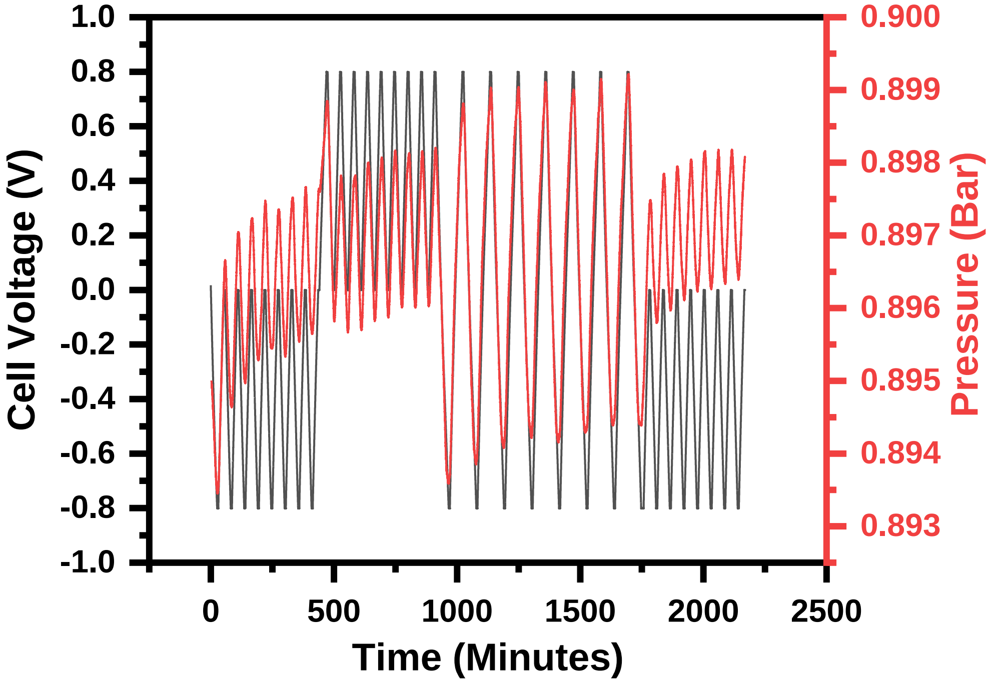

Figure S2: The effect of the change in voltage on the CO_2 pressure for a full SSA experiment including the negative, positive and switching charging protocols. The negative charging protocol was repeated at the end to ensure consistency. The gas cell used YP50F symmetric carbon electrodes with 1 M $Na_2SO_{4\ (aq)}$ electrolyte at 30 °C. Data in Figure 1 in the main text shows selected cycles from this dataset.

SI Figure S3

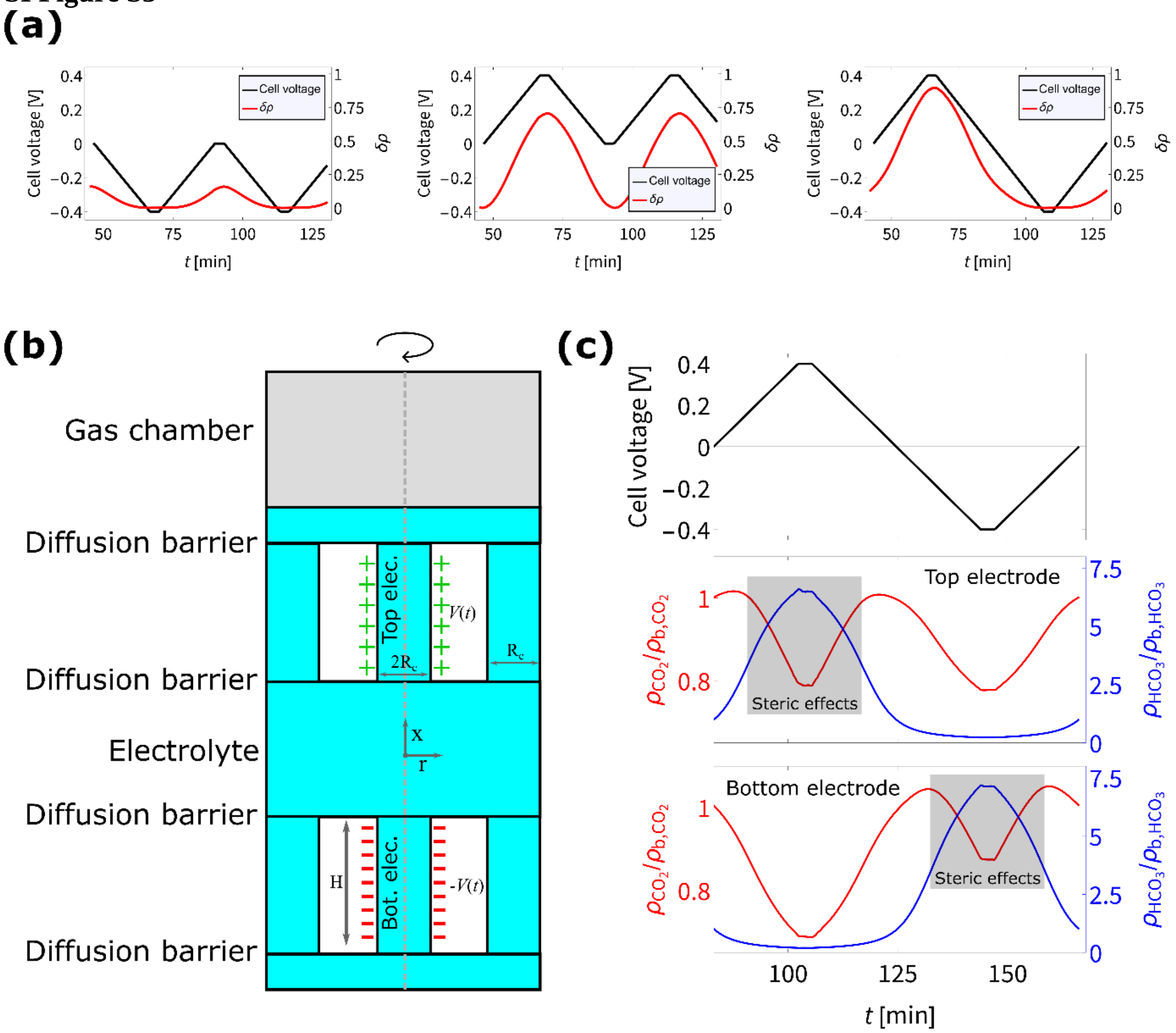

Figure S3: a) Finite-element calculations using COMSOL with the Steric effects term in Eq. 4 included that drives volume exclusion, showing the change in gas chamber CO_2 concentration during one switching protocol cycle when charged to ± 0.4 V with a voltage hold step of 5 minutes, b,c) the CO_2 (red) and bicarbonate ion (blue) concentration in the b) top and c) bottom electrode during the switching protocol cycle. Notes: The results in the main text do not include steric volume exclusion effects through the third term of Eq. 4, additionally the pH here is assumed to be 7 rather than 6. In Figure S3a we show $\delta\rho$ for one switching protocol cycle, showing a similar response compared to the results in Figure 2a, although with a larger total change in $\delta\rho$. In Figures S3b and S3c we show the CO_2 (red) and bicarbonate ion (blue) concentration in the b) top and c) bottom electrode during the switching protocol cycle. Here a distinct response is visible compared to the results in Figure 2c due to the presence of volume exclusion, highlighted in grey. As a voltage is applied, the charged particles accumulate inside the small pore and more favourably take up the limited pore space compared to CO2, which is not charged, creating the highlighted dip.

SI Figure S4

a)

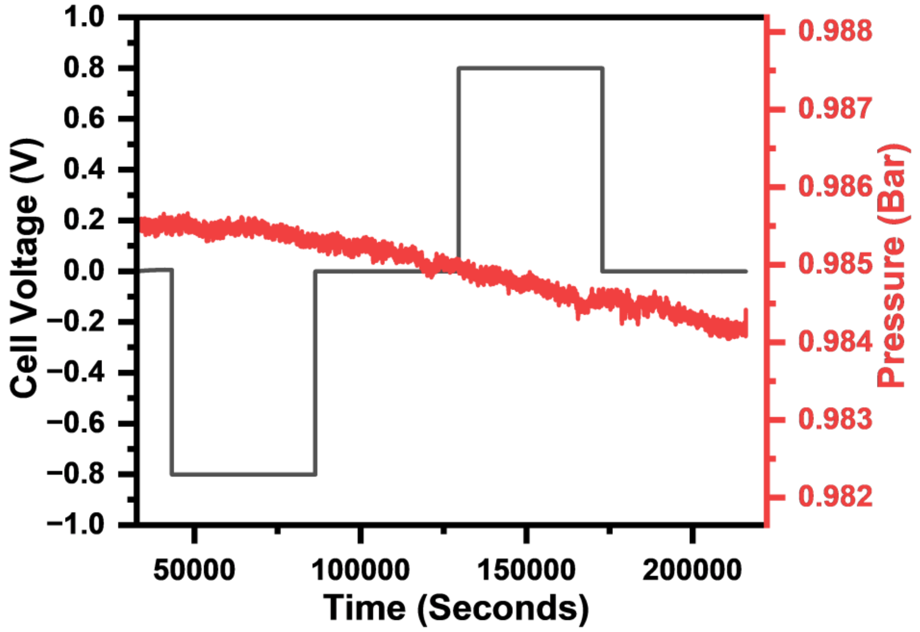

b)

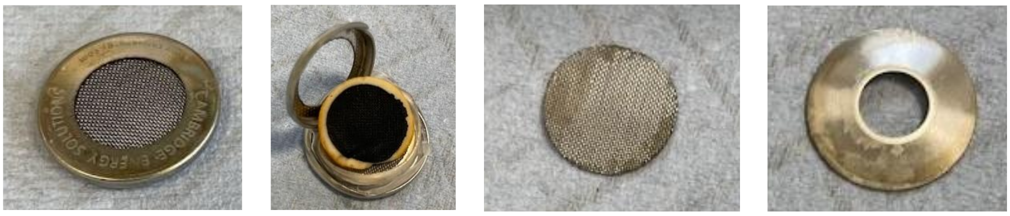

Figure S4: a) The cell voltage was charged to ± 0.8 V and held for 12 hours to observe the corrosion rate. This was conducted in a gas cell with no carbon electrodes to ensure only the corrosion was observed in the pressure data. This included the coin cell made up of the casing, spacers, spring and separator with 200 µL 1 M $Na_2SO_{4\,(aq)}$. b) Examples of the corrosion of the coin cell casing, the coin cell upon opening, the stainless steel mesh, and the stainless steel spring going left to right. This is the corrosion as a result of the experiments run in Figure S6. Notes: The pressure shown in Figure S4a is below atmospheric pressure and so the decrease shown is not due to a leak in the system and is instead from the presence of corrosion. This corrosion is commonly visually evident as shown in Figure S4b from the tarnish on the stainless steel components and from the orange precipitate on the separator.

SI Figure S5

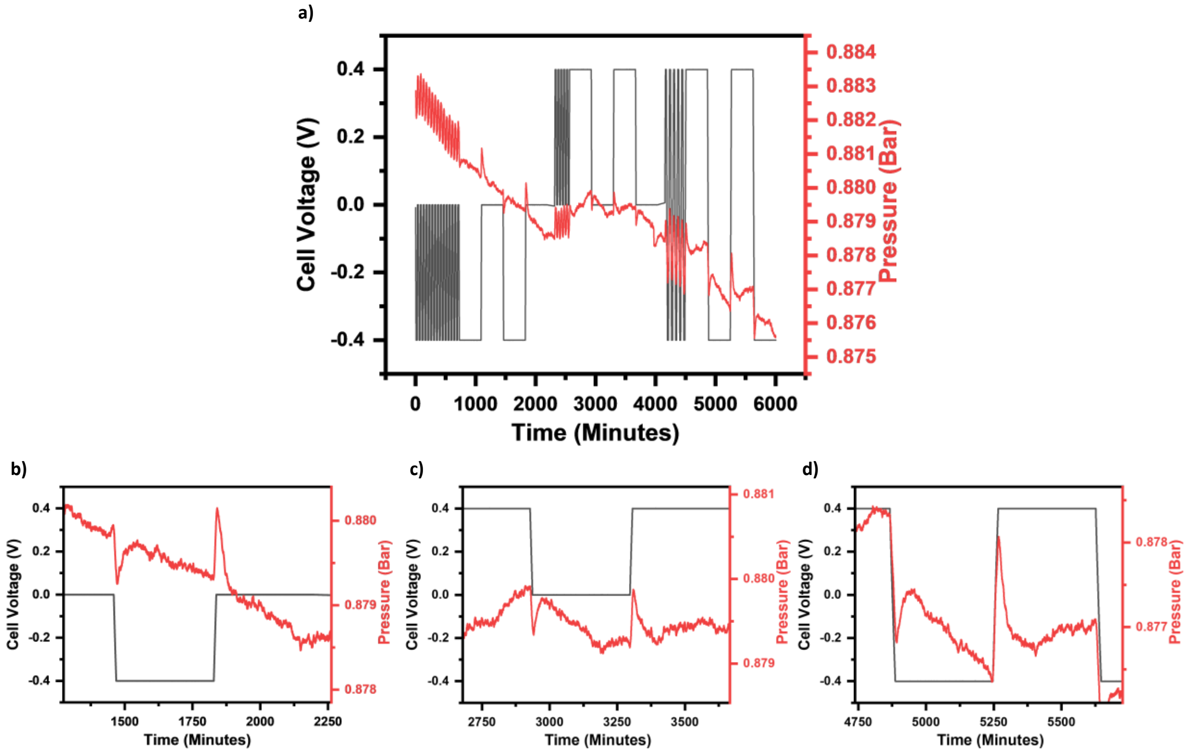

Figure S5: a) The full raw data set showing 6 hour cell voltage holds and zoomed in examples for b) the negative charging protocol, c) the positive charging protocol and, d) the switching charging protocol. The gas cell used YP50F symmetric carbon electrodes with 1 M $Na_2SO_{4\,(aq)}$ electrolyte at 30 °C. Notes: This is the un-correct data from the experiment shown in Figure 3a. The gas cell is kept at below atmospheric pressure to ensure that any gas leaks in the system would show as an increase in pressure (opposite to the trends observed for corrosion). Despite the noise seen in these experiments, with some instability in the pressure over time, we see that there is an obvious reversibility in the CO_2 pressure data. As the supercapacitor is charged in a negative direction (in both charging protocols), there is an initial capture of CO_2 but then a large proportion of this starts to desorb again at longer times. When the supercapacitor is charged in a positive direction, there is an initial release of CO_2 before the CO_2 begins to readsorb at longer times. This follows the same trends that we discuss in the main text in Figure 3a. Due to the noise in this experiment, it was repeated for a total of three times to ensure that the trends seen are accurate. These experiments are shown in Figure S6 and S7.

SI Figure S6

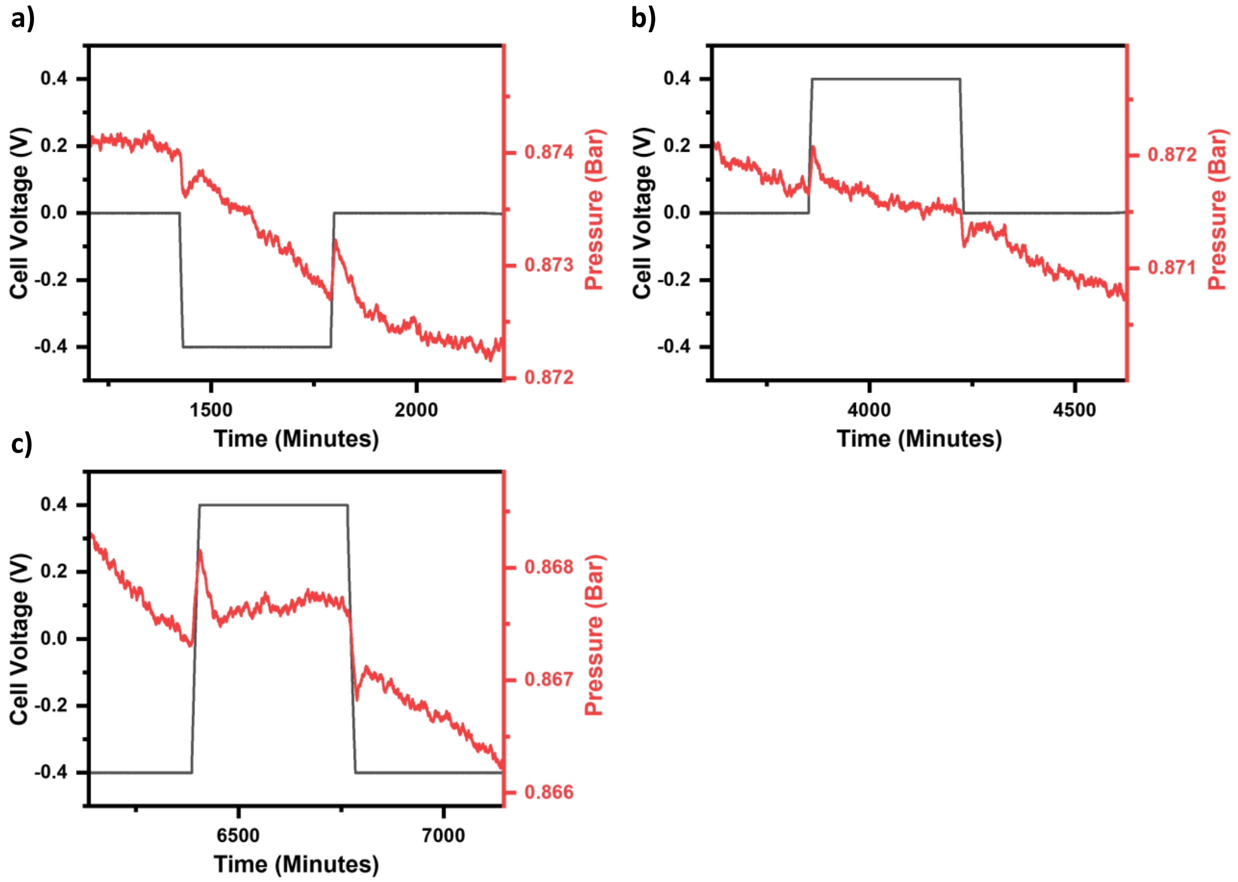

Figure S6: A second example for the purpose of reproducibility of the effect of a 6 hour cell voltage hold on the pressure for a) negative charging protocol, b) positive charging protocol and c) switching charging protocol. The gas cell used YP50F symmetric carbon electrodes with 1 M $Na_2SO_{4\,(aq)}$ electrolyte at 30 °C. Notes: This experiment is a repeat of the data shown in Figure 3a, S5, and S7 with a new gas cell to ensure reproducible trends are observed.

SI Figure S7

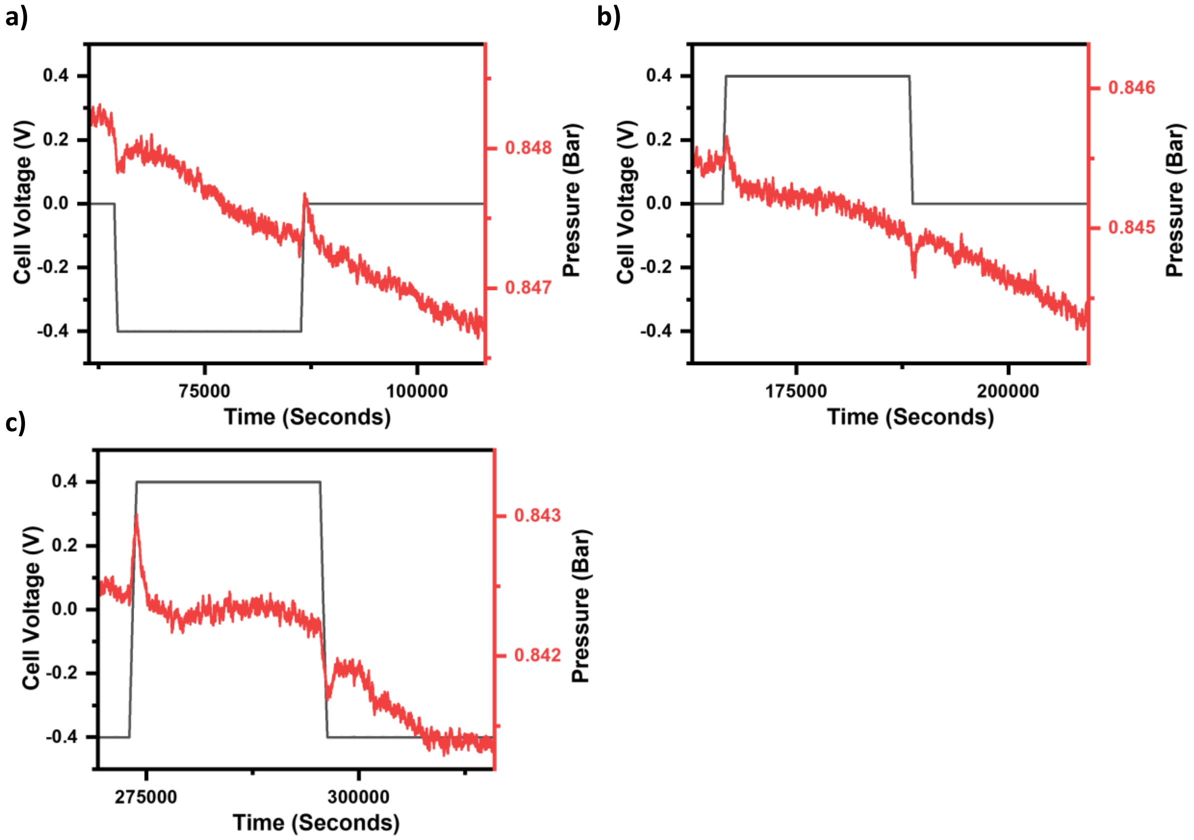

Figure S7: A third example for the purpose of reproducibility of the effect of a 6 hour cell voltage hold on the pressure for a) negative charging protocol, b) positive charging protocol and c) switching charging protocol. The gas cell used YP50F symmetric carbon electrodes with 1 M $Na_2SO_{4\,(aq)}$ electrolyte at 30 °C. Notes: This experiment is a repeat of the data shown in Figure 3a, S5, and S6 with a new gas cell to ensure reproducible trends are observed.

SI Figure S8

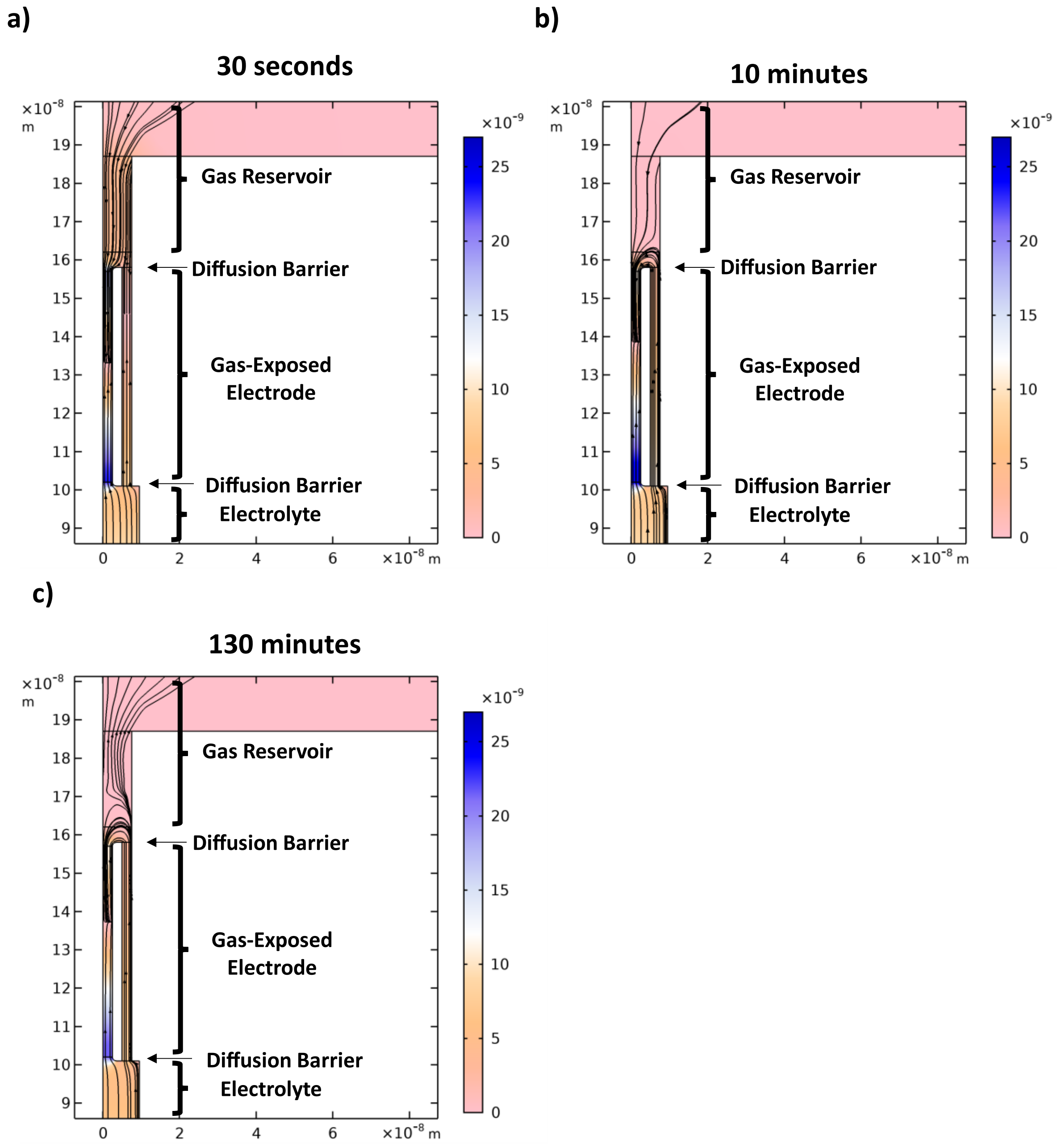

Figure S8: Snapshots of the CO_2 flux at the gas-exposed electrode in the model when the cell voltage reaches −0.4 V during a negative charging cycle when the charging times are fixed at a) 30 seconds, b) 10 minutes, and c) 130 minutes. These snapshots are collected from the simulations in Figure 3c in the main text. Colours underneath indicate the flux magnitude (mol m^{-2} s^{-1}), while the density of flux lines does not indicate flux magnitude quantitatively because additional lines are drawn to show all relevant fluxes in one figure. Notes: When the charging time is 30 seconds, CO_2 is still flowing into the gas-exposed electrode from the gas reservoir while the cell voltage is already at its minimal value of −0.4 V, indicating the system is mass transport limited. When the charging time is 10 minutes, three flux lines go into the gas-exposed electrode from the gas chamber, but most of the flux into the gas-exposed electrode is already coming from the bottom electrode indicating the system is close to its maximal adsorption capacity. When the charging time is 130 minutes, all flux lines into the top electrode are originating from the separator and bottom electrode, with some even going into the gas chamber showing that some reversibility occurs.

SI Figure S9

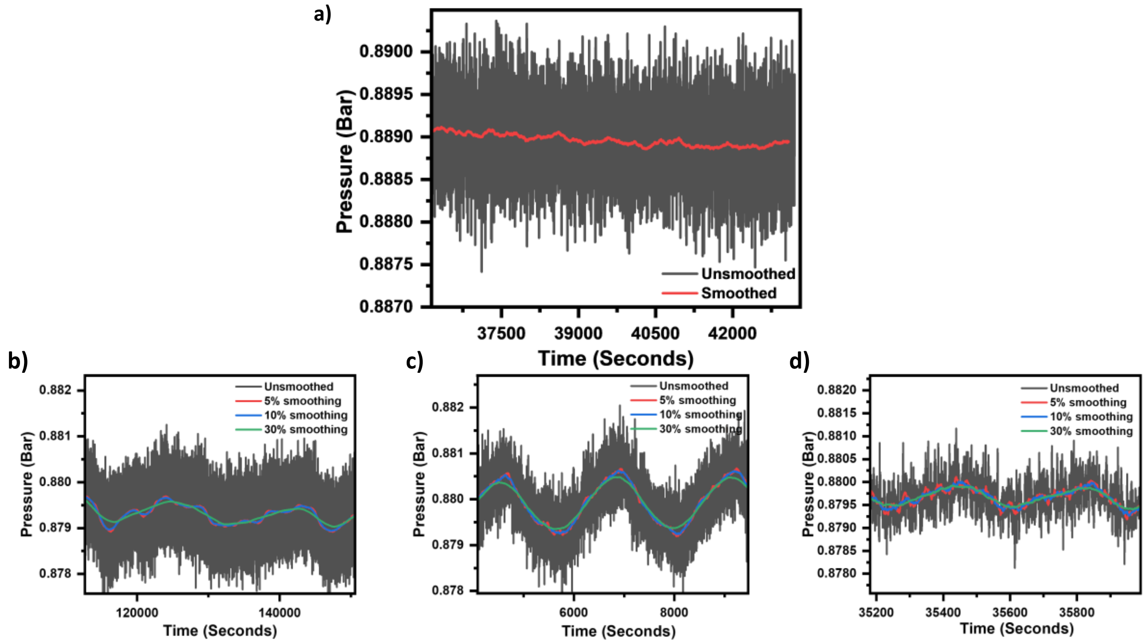

Figure S9: a) The effect of 10% data smoothing on a background recording of the pressure data when the cell had equilibrated in the oven at 30 °C for 10 hours. The effect of data smoothing on the trends that are extracted from the pressure data at different current densities: b) 5 mA g^{-1}, c) 30 mA g^{-1} and d) 150 mA g^{-1}. The same gas cell was used for all data sets with YP50F symmetric carbon electrodes with 1 M $Na_2SO_{4\,(aq)}$ electrolyte at 30 °C. Notes: 10% smoothing was chosen to ensure the pressure data is most accurately represented. Too much smoothing at 30% underestimates the magnitude of the amount of CO_2 captured by over smoothing the data. Conversely, undersmoothing the data with 5% smoothing means the trends in the pressure data are masked by the noise and finding the maximum (and minimum) is too hard to get accurate measurements. Therefore, 10% smoothing was chosen as the right balance between these two effects for all the pressure data shown.

SI Figure S10

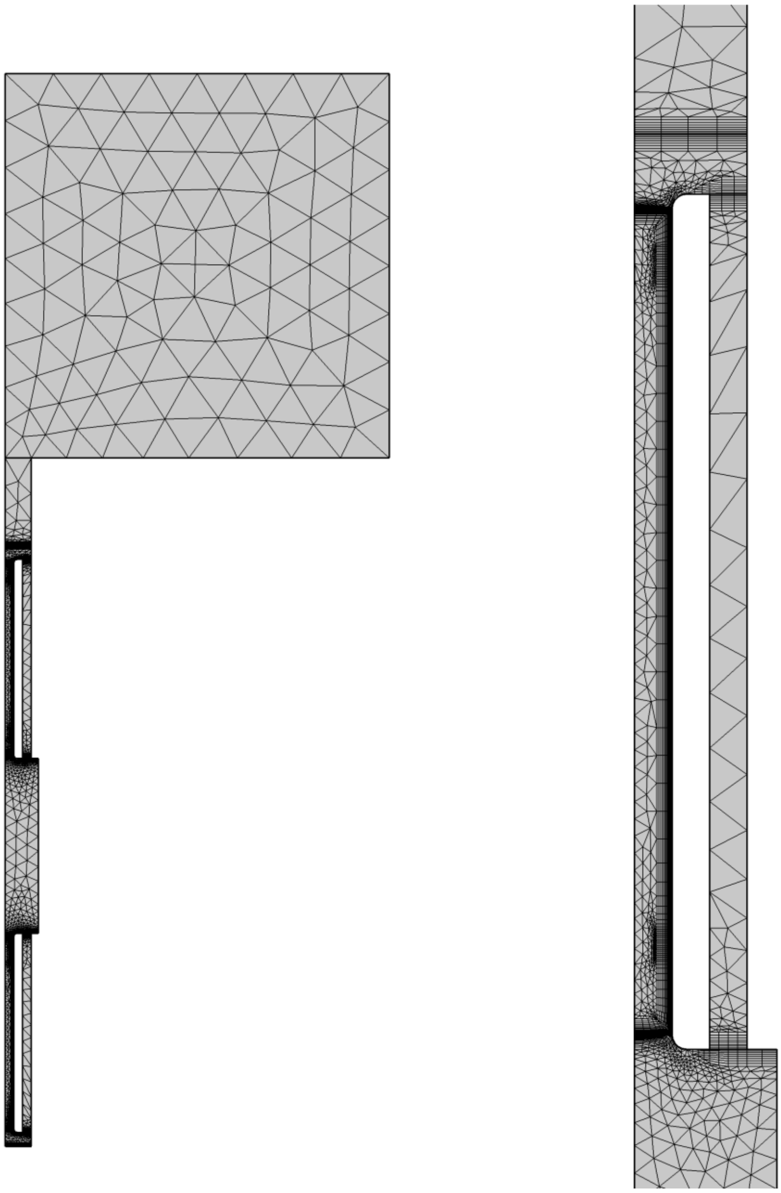

Figure S10: Screenshots of the mesh of the FE model schematically depicted on the left side in Figure 2(b). On the right side shows a zoomed in picture of the top-electrode mesh, showing the more precise elements at the charged surfaces and diffusion barriers.

SI Table

SI Table S1

Table S1: Further performance metrics for the experiment shown in Figure 1 and S1. It should be noted that coulombic efficiency cannot be calculated for the switching protocol due to charging both towards and away from zero during a single charging step so this is omitted.

Charging Protocol	Adsorption Capacity (mmol kg^{-1})	Coulombic Efficiency (%)	Resistance (Ω)	Gravimetric Capacitance (F g^{-1})	Electrical Energy Consumption (kJ mol^{-1})
Negative 1	60 ± 2	99 ± 2	5.6 ± 0.1	113 ± 4	52 ± 6
Positive	68 ± 2	97 ± 3	5.6 ± 0.1	115 ± 1	55 ± 5
Switching	150 ± 11	--	6.7 ± 0.2	137 ± 4	106 ± 13
Negative 2	60 ± 2	100 ± 1	5.8 ± 0.1	112 ± 1	50 ± 1
